# Oblique effects with multisegment spectacle lenses: 1. Images of a point object

**DOI:** 10.1111/opo.13469

**Published:** 2025-02-25

**Authors:** W. Neil Charman, David A. Atchison, Matt Jaskulski

**Affiliations:** ^1^ School of Health Sciences, Faculty of Biology, Medicine and Health University of Manchester Manchester UK; ^2^ Centre for Vision and Eye Research Queensland University of Technology Kelvin Grove Queensland Australia; ^3^ Indiana University School of Optometry Bloomington Indiana USA

**Keywords:** DIMS, multisegment, myopia control, spectacles

## Abstract

**Introduction:**

Multisegment (MS) spectacles are intended to slow myopia progression by modifying images falling on the peripheral retina. Some published optical treatments of these lenses assume normal incidence of light at the surfaces, but images falling on the peripheral retina are usually associated with oblique ray pencils. Here, we model representative images of point objects produced by the Hoya MiyoSmart MS spectacle lens when oblique ray pencils are used.

**Methods:**

Various imaging aspects of the MS lens alone and in combination with a suitable accommodating eye model for a 4D myope were evaluated using the Optical Design program Ansys Zemax OpticStudio. Configurations studied included object points at vergences of zero and –4 D, with the objects being either on the lens axis or at a field angle of about 30°. The effect on foveal vision of rotating the axis of the eye with respect to that of the lens was also considered. Images of point objects were described in terms of spot diagrams and fast Fourier transform point‐spread functions.

**Results:**

Symmetry and overall optical quality of images decreased with the obliquity of the ray pencils, due to the increased off‐axis aberrations of the lens and the eye. Images of near object points were strongly affected by the level of accommodation: optimal retinal image quality occurred when accommodation brought the carrier lens focus close to the retina, rather than that of the lenslets.

**Conclusions:**

Attempts to understand why MS lenses slow myopia progression need to consider the way in which through‐focus retinal image quality changes with obliquity of the ray pencils across the visual field and the possible effects of ocular accommodation.

**Key points**

- During MiyoSmart multisegment spectacle lens wear, images on the peripheral retina are usually formed by ray pencils which have passed obliquely through the lens and/or the eye.
- The off‐axis aberrations of both the lens and eye reduce the quality and contrast of images formed on the peripheral retina. The degradation increases generally with field angle, although the exact effects depend upon the eye's fixation direction with respect to the optical axis of the multisegment lens and the accommodation level of the eye.
- Rather than the carrier lens and lenslets giving two distinct planes of focus in the periphery, there is an extended region of approximate focus limited primarily by increasing astigmatism.
- Reduced image contrast in the periphery may play an important role in any myopia control effect produced by multisegment lenses.

## INTRODUCTION

Multisegment (MS) spectacle lenses show promise in reducing the commonly observed, but undesirable, age‐dependent increases in axial length and myopia in children by modifying the through‐focus image quality in the peripheral retina.^1^, ^2^, ^3^, ^4^, ^5^ Several designs are available, all based on a carrier lens with power to correct distance refractive error. Typically, the anterior lens surface is embossed with numerous small (around 1 mm in diameter), positively powered segments (lenslets) which cover about 40% of the carrier lens surface and produce multiple secondary non‐coaxial foci anterior to the retina.^6^, ^7^, ^8^ Thus, through‐focus optical image quality is asymmetrical about the carrier focus. It is hypothesised, mainly based on animal experiments, that this situation may slow ocular growth and myopia development.^9^, ^10^, ^11^, ^12^

In current practice, the central areas of the carrier lenses (about 10 mm in diameter) are left free of lenslets, as also may be the case in the outer parts of the lens. In the intermediate lenslet‐covered areas and in the absence of accommodation, the in‐focus retinal image produced by the carrier correction is reduced in contrast by the superimposed out‐of‐focus images created by the lenslets. The imagery in the more myopic focal plane corresponding to the lenslet focus is more complex. Each lenslet within the pupil forms its own focus, which is laterally separated from those produced by other segments. Thus, the overall retinal imagery depends upon the arrangement, sizes and separations of the segments, the pupil diameter and the gaze direction.

Some authors have presented calculations of the forms of the retinal point‐spread and modulation transfer functions produced by different designs of MS lens under specified conditions of pupil diameter and focus.^6^, ^7^, ^8^ However, these ‘nominal’ image characteristics assumed that the chief rays through the lenslets are parallel to the lenslet axes, whereas in practice, they become increasingly oblique to the axes in the more peripheral portions of the carrier lens.^3^ Moreover, in practical situations, the quality of the final retinal image will depend not only upon the aberrations of the carrier lens and lenslets but also those of the eye. Some of these off‐axis effects have been explored experimentally by Arias et al.,^13^ using a physical model with simulated average ocular aberrations and by Papadogiannis et al.^14^ using a double‐pass imaging system and wavefront measurements on adult eyes.

**FIGURE 1:**
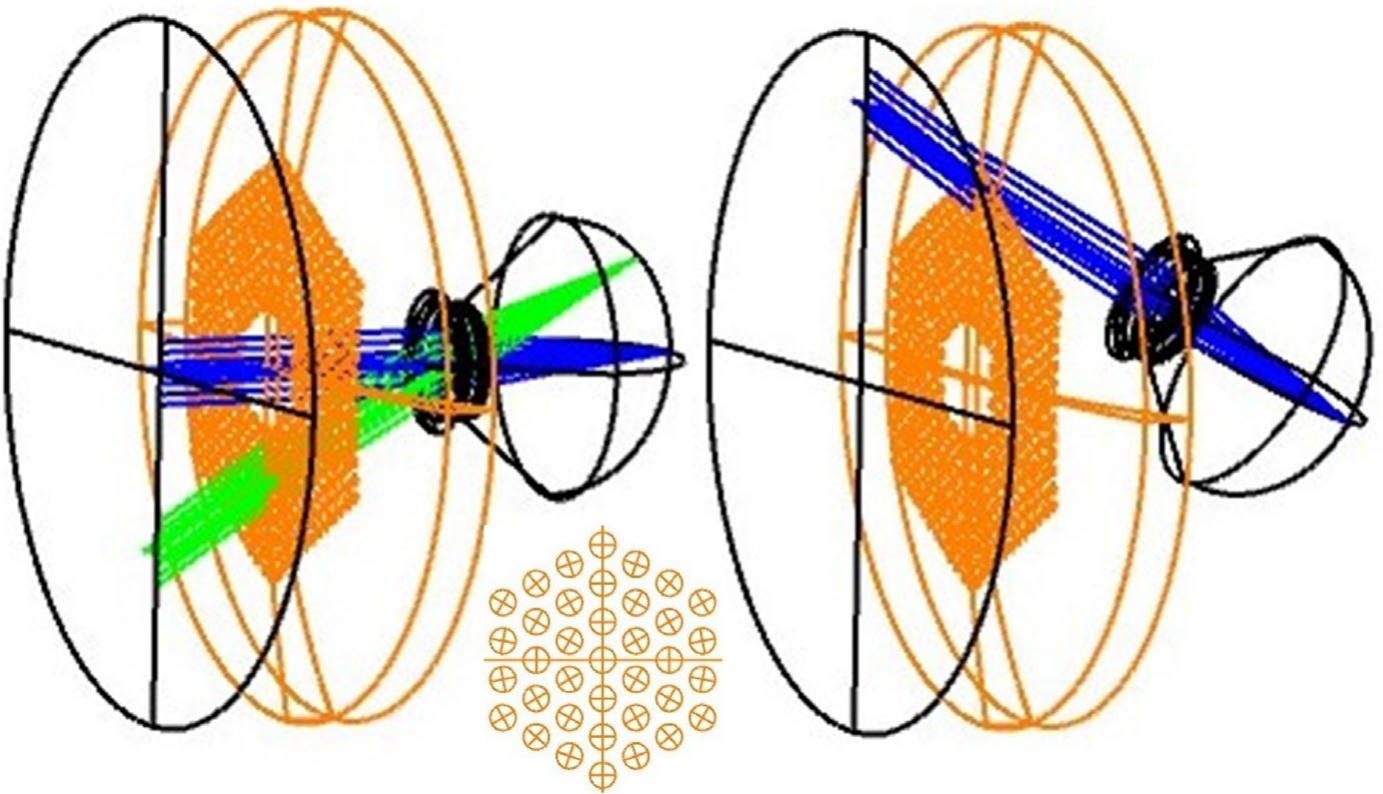
Orientation of rays and the lens. (Left) Carrier lens and eye optical axes coincide. Ray traces are shown for distance with both zero (blue) and 32.5° (green) field angles. Note that the −4 D power of the carrier lens affects the incidence angles of the rays at the cornea. (Right) Case where the eye rotates 31.7° to fixate a distant off‐axis object. The chief ray makes a non‐zero angle of incidence at the carrier lens surfaces and the −4 D power of the carrier lens affects the angles of the rays at the eye. The inset shows simulated lenslets—a central one surrounded by lenslets in three hexagons.

Even if the MS lens is fitted carefully, the effects will vary with the orientation of the axis of the eye with respect to the axis of the carrier lens, and the orientation of the object point with respect to the eye's axis. Examples are shown in Figure 1. In the left diagram, fixation lies on the optical axis of the carrier lens (blue rays), so that the chief ray for an axial fixation point passes along the common axes of the correcting lens and eye, that is, the angle of incidence of the chief ray is zero at both the lens surfaces and those of the eye. However, the obliquity of the ray bundles contributing to off‐axis retinal image points (green rays) increases with the field angle in all meridians.

In the right diagram, the eye is rotated to change fixation, so that its visual axis makes a non‐zero angle with the optical axis of the correcting carrier lens. The changes in the retinal point‐spread function will no longer be symmetrical about the visual axis. The exact effects will depend on numerous parameters, including the basic MS lenslet design and arrangement, the carrier power, the lens vertex distance, the optical characteristics of the eye and the direction and distance of the fixation point.

In general, it might be expected that obliquity of the chief ray for any ray pencil at the MS lens would degrade image quality due to the additional off‐axis aberrations introduced, particularly if the MS lenslets have highly aspheric forms, as in the Essilor Stellest (essilor.com/ca‐en/products/stellest/) design.^5^, ^7^ As noted earlier, in the case of the lens/eye combination, additional blur will arise from ocular aberrations. Inevitably, in most of the retina, the images during MS lens wear will be more blurred than those predicted on the basis of calculations of the axial images produced by the MS lenses alone.^6^, ^7^, ^8^ Further complications arise when the possible effects of accommodation are considered.

If we are to understand the possible modes of action of MS lenses in myopia control, some insight into the importance of these off‐axis and eye rotation effects is needed. Such insight should help to answer questions such as whether it is the presence of a second, myopic plane of focus with higher spatial frequency content or the blur and contrast loss in the lenslet images which is more important for any control effect produced.

The present paper supplements earlier experimental work^13^, ^14^ by exploring theoretically some typical examples of the impact of factors such as object direction and distance, and the eye's aberration, accommodation and rotation on the retinal point‐spread functions found with the earliest of the MS lenses, the Hoya Corporation MiyoSmart (hoyavision.com/vision‐products/miyosmartVr25). Hoya refers to the MS technology as defocus incorporated multiple segments (DIMS).^1^, ^2^ Calculations using Zemax procedures were carried out on imagery using the MS lens alone or the MS lens in combination with an accommodating model eye.

## METHODS

### MS lens

The Hoya MiyoSmart lens has nominally +4.0 D circular lenslets of 1.0 mm diameter, arranged in a triangular lattice of side length 1.5 mm, that is, the centre‐to‐centre distance is 1.5 mm.^7^ This lattice extends over an area of the carrier lens with inner and outer hexagonal boundaries which are perpendicularly separated by 9.4 and 33.0 mm, respectively. Thus, the complete arrangement consists of nine nested hexagons of lenslets. For the present purposes, we give the smallest hexagon the designation H1, the largest H9. The centres of the corner lenslets of H1 lie at 6.0 mm from the carrier lens centre, for H2 at 7.5 mm, etc., up to 18.0 mm for H9. It was assumed that these distances are measured along the surface rather than in a plane, and that hexagons are aligned vertically (Figure 1). To simulate the case when incidence is normal and to allow comparison with earlier published results,^6^, ^7^, ^8^ in some calculations, four additional smaller hypothetical hexagons were considered, comprising a single lenslet at the centre of the carrier surrounded by three neighbouring hexagons (see inset, Figure 1). With this modelling arrangement, the triangular lattice of lenslets covered the entire central area of the carrier, with no central clear aperture.

The lenses are made of polycarbonate (refractive index 1.586), and the carrier lenses have a +3.0 D base curve when measured with a Geneva lens measure calibrated for 1.523 index. The true front surface powers and radii of curvature are 3.36 D and 174.33 mm, respectively. For this work, a lens back vertex power of −4.00 D was assumed. For a 1.0‐mm central thickness, this gives a back surface radius of curvature of 173.96 mm.

The assumed anterior surface shapes of the lenslets were based on the work of Gantes‐Nuñez et al.^7^ The latter determined the aberrations of lenslets, using plano distance‐powered carrier lenses. Wave aberrations were measured with a single‐pass Hartmann–Shack device (SHS Ophthalmic base, Optocraft GmBH, optocraft.de/en/) designed for use with contact lenses. Using only the rotationally symmetrical aberration coefficients up to the sixth order, the wave aberration is given by

$$\begin{array}{c} W\left(r\right)=\sqrt{3}c_{2}^{0}\left(\frac{2r^{2}}{R^{2}}-1\right)+\sqrt{5}c_{4}^{0}\left(\frac{6r^{4}}{R^{4}}-\frac{6r^{2}}{R^{2}}+1\right)\\ \;+\sqrt{7}c_{6}^{0}\left(\frac{20r^{6}}{R^{6}}-\frac{30r^{4}}{R^{4}}+\frac{12r^{2}}{R^{2}}-1\right) \end{array}$$

where $c_{2}^{0}$, $c_{4}^{0}$ and $c_{6}^{0}$ are defocus, spherical aberration and secondary spherical aberration coefficients, respectively; *r* is distance from pupil centre; and *R* is stop semi‐diameter.

The corresponding power across the pupil

$$F\left(r\right)=\frac{\mathrm{dW}}{\mathrm{dr}}/r$$

is given by

$$\begin{array}{c} F\left(r\right)=4\sqrt{3}c_{2}^{0}/R^{2}+12\sqrt{5}c_{4}^{0}\left(2r^{2}-R^{2}\right)/R^{4}\\ \;+24\sqrt{7}c_{6}^{0}\left(5r^{4}-5r^{2}R^{2}+R^{4}\right)/R^{6} \end{array}$$

The power corresponding to the centre, that is, for *r* = 0, is

$$F\left(0\right)=\left[4\sqrt{3}c_{2}^{0}-12\sqrt{5}c_{4}^{0}+24\sqrt{7}c_{6}^{0}\right]/R^{2}$$

This was taken as the addition power of the lenslet and used to determine its radius of curvature. Gantes‐Nuñez et al.^7^ found that the coefficients of all hexagons of the Hoya lenses were similar. Across a 1.35‐mm diameter region, these were $c_{2}^{0}$ = +0.1452 mm, $c_{4}^{0}$ = −0.0748 mm and $c_{6}^{0}$ = −0.01142 mm (unpublished data). Substituting these values into the final equation gives *F*(0) = 4.050 D, which in turn gives a lenslet radius of curvature as 586/(3.36 + 4.05) = 79.1 mm, with +3.36 D being the front surface power as determined above.

**FIGURE 2:**
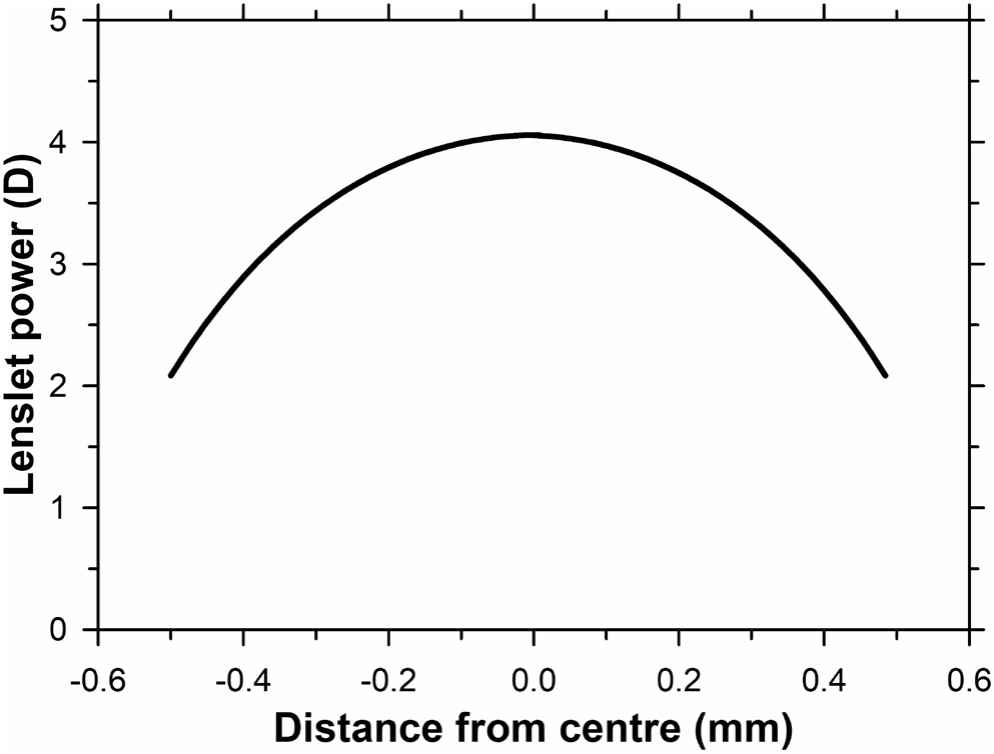
Hoya MiyoSmart (hoyavision.com/vision‐products/miyosmartVr25) lenslet power as a function of the distance from the lenslet centre. The surface shape is described by the surface sag equation $z=\frac{r^{2}}{R+\sqrt{R^{2}-\left(1+Q\right)r^{2}}}+A_{6}r^{6}$ where *r* is the distance from the lenslet centre, *R* is the radius of curvature in the centre (79.0716 mm), *Q* is conic asphericity (−10,930) and *A*_6_ is a sixth‐order coefficient (−0.00338 mm^−5^).

Using the Optical Design program Ansys Zemax OpticStudio R2.01 (www.ansys.com/products/optics/ansys‐zemax‐opticstudio), the negatives of the coefficients were included as a Zernike phase surface in combination with a lenslet having the radius of curvature described above and a plano lens. On the basis of the interferometric measurements of Radhakrishnan et al.,^8^ lenslets thicknesses were set as approximately 0.0015 mm. In an optimisation, conicoid asphericity *Q* and the sixth‐order coefficient *A*_6_ of the lenslet were set as variables. The Zernike phase surface was removed, and the power determined across the aperture. The present results for the Hoya lenslets (Figure 2) drop a little more slowly with radial distance than those given by Gantes‐Nuñez et al.,^7^ for example, power at the edge is 2.1 D rather than the 1.5 D reported earlier (see their Figure 5 left, wavefront vergence for radial co‐ordinates ±0.5 mm).

### Model eye

The model eye used was an accommodating version of the earlier Atchison model eye,^15^, ^16^ in which certain parameters depend linearly on the spectacle refraction, including anterior corneal radius of curvature, vitreous depth, and vertex radii of curvatures and asphericities of a bionic retina. In the accommodating eye, relevant lens‐related parameters were varied with accommodation. For the illustrative purpose of the present study, the spectacle refraction used to derive the parameters of the model eye was taken as –4.00 D, matching the power of the carrier in the MS lens.

### General configuration of the lens/eye system

The stop was set to 5.0 mm diameter, on the assumption that we are dealing with large pupils in children. The stop (eye pupil) was assumed to be 15.7 mm from the back surface of the lens, and the back vertex distance of the lens was 12 mm. Chosen object points were in a plane either at infinity (‘distance’) or 250 mm from the lens front surface (‘near’). Thus, with the distant object point, the image formed by the carrier lens should nominally lie on the retina, and in the absence of accommodation, the near object point should be imaged on the retina by the lenslets.

### Optical modelling and ray tracing

The Optical Design program Ansys Zemax OpticStudio R2.01 was used to evaluate various aspects of the MS lens and MS lens/eye systems. Images of point objects were determined either by passing a matrix of rays through the system using geometrical optics, from which the intersections of the rays with the chosen image surface could be determined and hence the spot diagram (SD) could be generated, or by calculating fast Fourier transform point‐spread functions (PSF). Unlike the SDs, the PSFs, which are mapped on planes centred on and perpendicular to the chief rays, include the effects of diffraction. In both cases, a wavelength of 550 nm was used.

For stationary vision, in which the optical axes of the carrier lens and eye remained coincident, field angles in object space were chosen so that chief rays always passed through the centre of one of the lenslets. Similarly, for ray tracing involving the rotating eye and foveal vision, eye rotation angles were again chosen so that chief rays passed through the centres of lenslets.

Modelling could be done for the lens with or without the eye. The centre‐of‐rotation was modelled in Zemax as a ‘co‐ordinate break’, 27 mm behind the back surface of the lens, about which the rest of the optical system could be rotated, in *x* and *y* directions as appropriate. Without the eye, a stop was placed 11.86 mm in front of the centre of rotation, at the location of the entrance pupil of the model eye (15.14 mm behind the lens), and the image surface was set at the paraxial focus for a distance object. The image surface was concentric at the centre‐of‐rotation for foveal vision with the rotating eye and concentric at the stop for stationary, peripheral vision. This approach is meaningful for a lens by itself for foveal vision as the power errors are those experienced by the eye. Although this approach has been used for peripheral vision, it must be kept in mind that the ideal image surface depends upon the optics of the eye including the retinal shape.

With the eye, the stop was placed 11.30 mm in front of the centre‐of‐rotation (15.70 mm behind the lens). This required that, in Zemax, ray tracing proceeded through the MS lens to the centre‐of‐rotation, then backwards 15 mm to the cornea, and then forwards through the cornea to the stop at the front of the lens, through the crystalline lens with a gradient index, and to the retina. For the near object points, the images were calculated first with an unaccommodated model eye and then for an eye with an accommodation response which matched the accommodative demand (+4 D).

The MS lens was modelled in Zemax as a ‘non‐sequential’ component. The hexagons were modelled using the ring array object feature where a parent object (a lenslet) is rotated outwards along an arc, corresponding to the front surface of the lens, and then rotated about the optical axis in steps corresponding to the locations of the lenslets. The six corners of the inner hexagon were modelled first, followed by the adjacent spots in the hexagon, and so on for a total of four arrays. Attention was then given to the next layer of lenslets, and so on, for a total of 72 arrays across nine hexagons.

## RESULTS

As noted earlier, in all calculations which included lenslet‐covered areas of the MS lens, the imaging pencils were positioned so that the central chief ray passed through the centre of a lenslet. This allows easy comparison of images. In practice, of course, such alignment does not usually occur and any lateral movement of the ocular pupil with respect to the lens will result in ray pencils passing through a different area of the lenslet array, with consequent effects on the form of the images. We consider first some examples of the imagery obtained with the lens alone and then effects with the complete lens–eye system. Results are for on‐axis positions or for off‐axis positions along the vertical meridian.

### Lens alone

#### Non‐rotated MS lens alone with clear central area: On‐axis object point, distance and near

**FIGURE 3:**
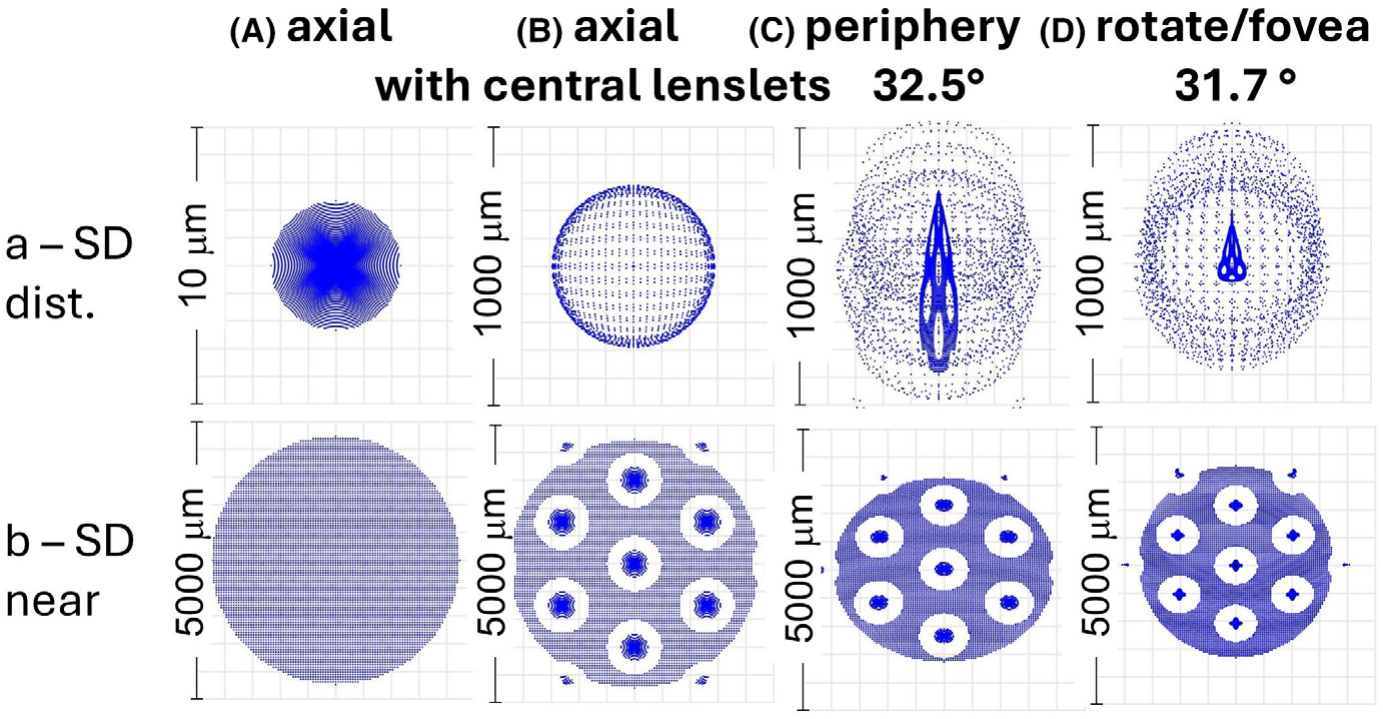
Spot diagrams (SDs) for the lens alone. (A) Axial object points for a carrier lens with no lenslets; (B) axial object points with simulated central lenslets; (C) 32.5° field; (D) foveal vision (rotation angle 31.7°). (a) Distance object; (b) near object. Note the variations in scale.

With the unmodified MS lens alone, the best image is obtained when the object point is distant and placed on the optical axis of the lens. It is imaged through the clear, lenslet‐free, central area of the lens. Figure 3A(a) shows the SD for this configuration and Figure 3A(b) shows the image where the object point is at a distance of 250 mm. Note the compactness and rotational symmetry of the far image, which in monochromatic light approximates to a point (SD) since the carrier lens on‐axis monochromatic aberrations (spherical aberrations and defocus) are very small. The near image approximates to a blur circle. These ‘optimal’ images (which would also be given with conventional single‐vision lenses) form a standard against which other images can be judged.

#### Non‐rotated MS lens alone with simulated central lenslets: On‐axis object point, distance and near

As discussed under ‘Methods’, four additional hexagons of lenslets were added to the centre of the carrier lens so that the case considered by earlier authors,^6^, ^7^, ^8^ in which the chief ray is incident normally on the lenslets, could be simulated (Figure 1). The SDs obtained by tracing an array of rays from an object point on the optical axis of the carrier at either infinity or at a distance of 250 mm through the lens alone are shown in Figure 3B(a,b). The forms of the images are very similar to those published by earlier authors.^6^, ^7^, ^8^ In Figure 3B(a), with a lateral extent much greater than in the corresponding lenslet‐free case of Figure 3A(a), the distant SD consists of a central bright core, attributed to the carrier lens, surrounded by a broad blur patch comprised of the superimposed blur circles formed by the lenslets. However, this SD gives a poor representation of the high central concentration of the superimposed rays of the carrier lens component since the central spots overlap. Note that the circular blur patch due to the summated effects of the lenslets has a bright border. This is caused by the negative spherical aberration of the lenslets (see Figure 2).

In the much larger SD for the axial ‘near’ image (Figure 3B(b)), the laterally separated lenslet images are obvious and are seen against the broad patch of defocused light due to the carrier lens.

#### Non‐rotated MS lens alone: Off‐axis object point, distance and near

The images change markedly when off‐axis imagery is considered. Figure 3C shows distance and near results when the chief ray passes through hexagon H3, corresponding to a vertical field angle of 32.5°. In the distance case, instead of the carrier forming an almost point‐focus, lenticular off‐axis astigmatism and coma have considerably blurred the SD (Figure 3C(a)). The lenslets again form blur ‘circles’, but these are slightly elliptical and vertically displaced with respect to one another. Interestingly, the effects of an off‐axis observation appear to be relatively less important when the object point is near (Figure 3C(b)) than in the distance case. Defocus appears to dominate other aberrations. The component blur circles are slightly flattened in the meridian of the field angle.

#### Rotated MS lens: Distant and near object points

Here, the object points initially lie on the optical axis of the MS lens. The stop and image surface are then rotated about the eye's centre of rotation (with the lens remaining stationary) through an angle such that the chief ray passes through the 8th hexagon of lenslets (H8): the rotation angle is 31.7° and the object field angle is 35.6°. The distance SD (Figure 3D(a)) has a similar basic form to the non‐rotated cases (Figure 3B(a)), but the image is no longer rotationally symmetrical. Distorted versions of blur patches formed by the carrier and lenslets can, however, be recognised. The carrier lens is fairly well corrected for off‐axis vision. although some influence of coma is seen (compare with Figure 3B(a)).

When the corresponding images for a near object point are considered (Figure 3D(b)), there are only minor differences from those for a non‐rotated lens.

### Combined lens and eye

As discussed earlier, when the eye is included, the possibility of accommodation by young lens wearers must be considered. For simplicity, we assume either that there is no accommodation for both distant or near object points, that is, that the eye is fully presbyopic, or that there is no accommodation to the distant point but accurate accommodation to the near object (i.e., that 4 D of accommodation is exercised). The sections below have the same sequence as for the lens alone. Both SDs and fast Fourier transform PSFs are presented. In angular terms, 100 μm corresponds to a subtense of about 20 min of arc, with the exact value depending upon the visual field angle.

#### Non‐rotated MS lens and eye: On‐axis object point, distance and near

Here, the eye views through the central, lenslet‐free area of the lens, which effectively acts as a single‐vision lens as far as foveal vision is concerned.

**FIGURE 4:**
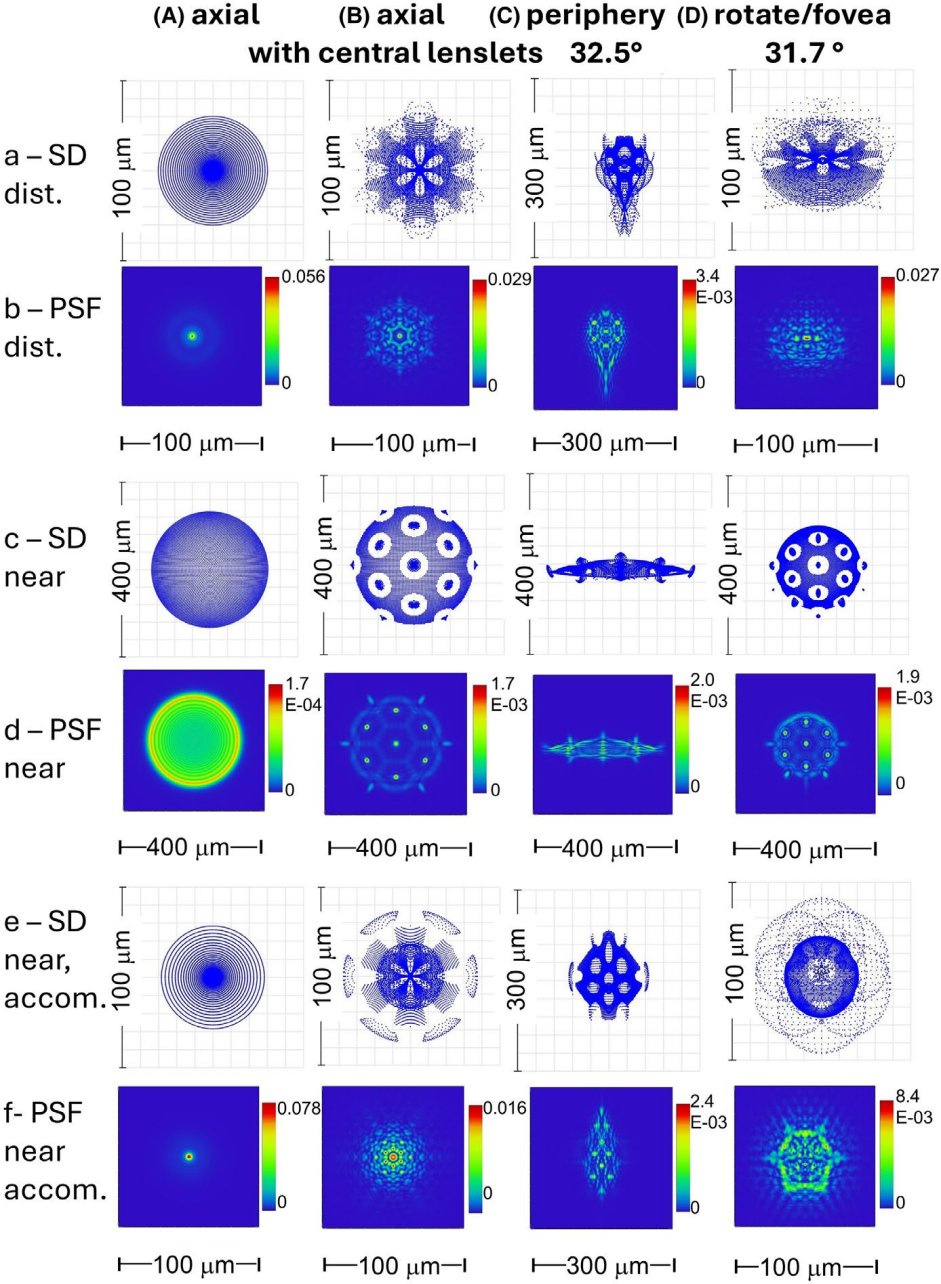
Spot diagrams (SDs) and point spread functions (PSFs) for the lens and eye combination. (A) axial object points for a carrier lens with no lenslets; (B) Axial object points with simulated central lenslets; (C) 32.5° field; (D) Foveal vision—rotation eye angle 31.7°. (a) SDs for distance object; (b) PSFs for distance object; (c) SDs for near object; (d) PSFs for near object. (e) SDs for near object with accommodation; (f) PSFs for near object with accommodation. Note the variations in scale.

Figure 4A shows the SD and PSF for distant (a,b) and near (c,d) point objects when there is no accommodation and Figure 4A(e,f) illustrates those obtained in the near case when the eye accommodates appropriately. The distant images are very compact, since the only sources of degradation are diffraction and the higher order aberrations of the carrier lens and eye. The accommodated near images are also compact. Not surprisingly, the MS lens wearer viewing through the central region of the lens with active accommodation should experience good foveal vision at all normal working distances.

#### Non‐rotated MS lens with simulated central lenslets and eye: On‐axis object point, distance and near

Four additional hexagons of lenslets were added to the centre of the carrier lens to give simulation of normal incidence at the lenslets. Figure 4B(a–d) shows axial images for the lens‐unaccommodated eye combination. In the distance case (Figure 4B(a,b)), the image is more complex than in the corresponding lens‐only case (Figure 3B(a)). The image is compact, but the PSF (Figure 4B(b)) reveals the presence of strong diffraction effects. In contrast, the image (Figure 4B(c,d)) for the near axial object point differs little from that of the lens alone, apart from some sagittal compression (compare with Figure 3B(b)).

If the eye accommodates to the near target, the SD and PSF (Figure 4B(e,f)) are almost the same as the distance images shown in Figure 4B(a,b), with the minor variations being attributable to changes in ocular spherical aberration. Thus, accommodation produces a more compact image.

#### Non‐rotated MS lens and eye: Off‐axis object point, distance and near

Figure 4C(a,b) shows the retinal image of a distant point at a field angle of 32.5° when the axes of the MS lens and eye are coincident. The SD (Figure 4C(a)) and PSF (Figure 4C(b)) are similar in appearance and are larger and more complex than when the object point is on‐axis, with a marked extension in the radial direction.

When the off‐axis near object point is observed with an unaccommodated eye, the image is strongly compressed along the direction of the field meridian, although features due to the lenslets and carrier can be identified (Figure 4C(c,d)). However, if the eye accommodates by 4 D, the off‐axis image becomes more compact (Figure 4C(e,f)).

#### Rotated lens/eye system: Distant and near object points

Here, the object is moved away from the lens axis such that, for foveal viewing, the eye is rotated 31.7° and the chief ray passes through the corner lenslet of the 8th hexagon (H8). Figure 4D(a,b) shows that the image of the distant point remains reasonably compact after the rotation. The spot diagram and PSF are broadly similar. In comparison with the axial cases without or with simulated central lenslets in Figure 4A(a,b),B(a,b) the image lacks rotational symmetry and is compressed in the sagittal direction.

When the object distance from the lens is reduced to 250 mm, the images for the unaccommodated eye in Figure 4D(c,d) differ only slightly in terms of their rotational symmetry from those in the corresponding axial case with simulated central lenslets in Figure 4B(c,d). However, if the eye accommodates by 4 D, the image of the off‐axis near object point again becomes more compact as shown in Figure 4D(e,f). As in the distance case, the accommodated near image is still worse than would be obtained axially with a real multisegment lens with a central clear aperture (Figure 4A(e,f)).

### Through‐focus evolution of the retinal image

**FIGURE 5:**
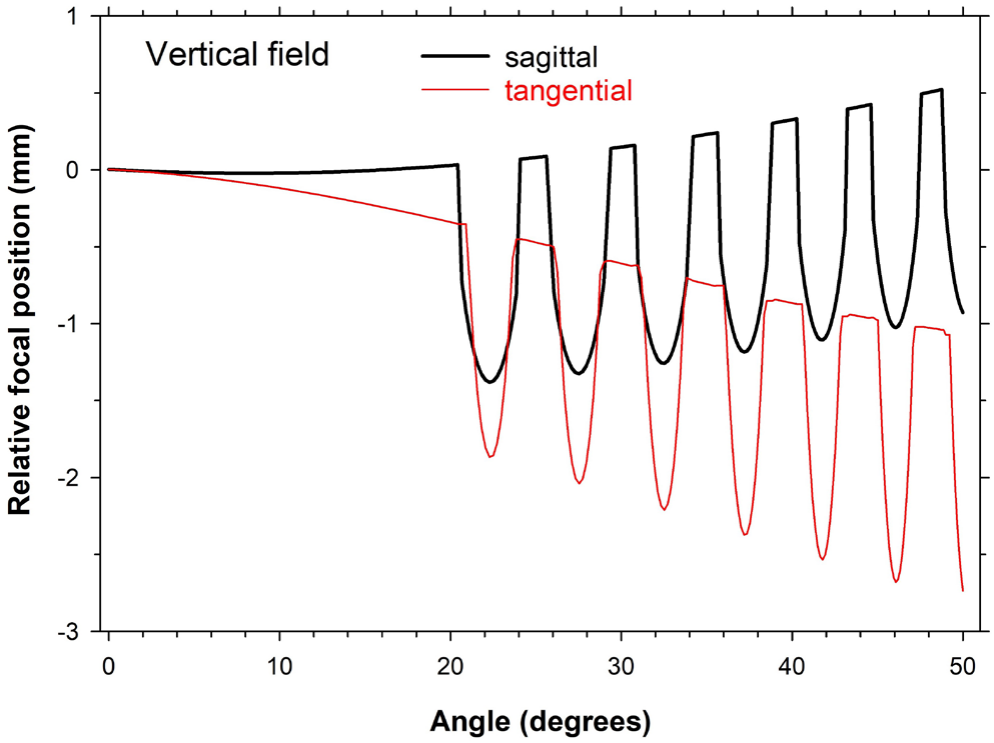
Positions of sagittal and tangential foci relative to the retina as a function of vertical field angle, for a distant object field. Tangential foci correspond to the refraction being in the plane that contains field points and chief rays; the sagittal foci correspond to refraction in the orthogonal meridian.

The centres of the preceding lens/eye images (Figure 4) were for specific positions of focus, that is, where the chief rays intersected the surface of the retina of the assumed model eye. If different image surfaces are considered, the form of the spot diagram changes. This raises the question of the way in which the distance between local focus of the ‘image’ and the retina varies as a function of retinal position or field angle. As an example, Figure 5 shows the position of the in‐focus image surface in relation to the retina, as measured along the chief ray in the case of a lens‐unaccommodated eye system in peripheral vision as a function of the field angle.

**FIGURE 6:**
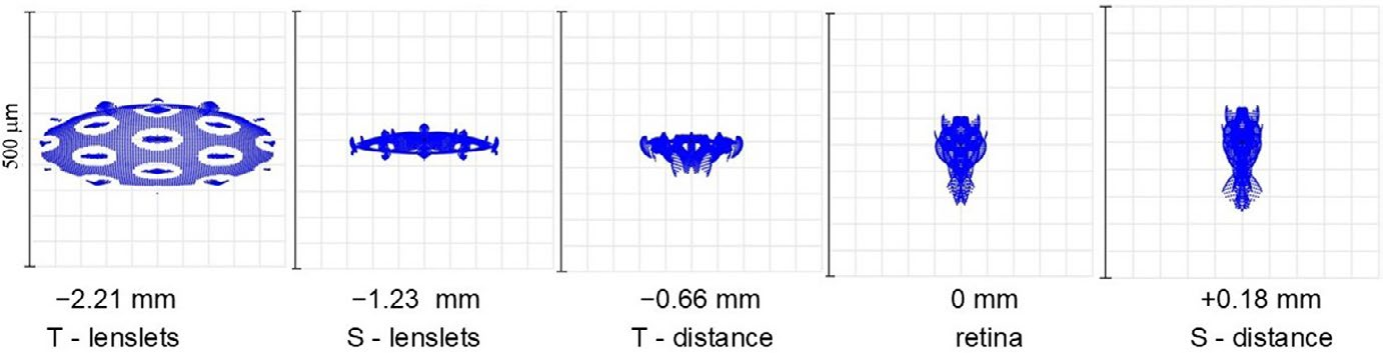
Tangential (T) and sagittal (S) images of distant and near object points formed in peripheral vision for a vertical field angle of 32.5°. The ‘far’ sagittal and tangential planes are +0.18 and −0.66 mm, respectively, from the retina along the chief ray (equivalent to +0.50 and −2.15 D) and the ‘near’ planes are −1.23 and −2.21 mm along the chief ray (−4.21 and −7.71 D).

In relation to the retina, a variety of ‘image’ surfaces can be identified, including the sagittal and tangential surfaces resulting from the oblique astigmatism of the lens–eye system. Figure 6 shows the appearance of the SD when focus for a lens/eye system having a common axis is set to values appropriate to the sagittal and tangential image surfaces when distant and near objects points are observed at a vertical field angle of 32.5°. This corresponds to the chief ray passing through hexagon H3. While component blur patches associated with the carrier and lenslets can be recognised, the forms of the overall image appear to be dominated by oblique astigmatism.

## DISCUSSION

The present calculations for the forms of the images of distant and near axial object points given by the MiyoSmart lens alone are similar to those found by earlier authors.^6^, ^7^, ^8^ In general, the contributions of the carrier lens and lenslets are easily distinguishable. Images formed by the lens alone or on the fovea of an eye whose axis coincides with the lens axis are similar in form. However, if an object point does not lie on the common axis of the lens and eye, or the eye turns away from the lens axis to view a non‐axial object point, major changes may occur in the form of the retinal images, particularly for distant object points (see Figures 3 and 4). It cannot be assumed that local retinal imagery is the same as that calculated for the axial case, and under most circumstances, the image quality will be worse. The physical images simulated by Arias et al.^13^ and the visual measurements by Papadogiannis et al.^14^ for MiyoSmart lenses led to similar conclusions. The oblique astigmatism (relative peripheral refractive error) of the eye makes an important contribution to image degradation in many circumstances.

The present calculations do not include the effects of chromatic aberration, which will degrade the retinal images further under polychromatic conditions. A transverse shift of the ocular pupil with respect to the lenslet array, which results in the chief ray no longer passing through the centre of a lenslet, will produce related transverse shifts in much of the fine structure of the SDs and PSFs. Modulation transfer functions and simulated images can be derived from the point images by respectively taking Fourier transforms of the image PSFs and by convoluting the object's light distribution with the PSF.

Given the imaging properties of the MiyoSmart lens, how can we account for their apparent ability to slow the rates of progression of myopia and axial length in children? In any discussion of the mode of action of MS spectacles, two interesting initial questions arise concerning the visual behaviour of children who wear them. Do all children always direct their eyes so that their fixation axes pass through the lenslet‐free central areas of the lenses, turning their heads to view initially off‐axis objects, and do they accommodate in the same way as they would with a single‐vision correction?

No systematic studies appear to have been carried out to explore either issue. Fixating through a more peripheral, lenslet‐covered area of the lens will always reduce the contrast of the retinal image and the visual acuity achieved,^17^ as compared with fixating through the central clear area, so that the latter is likely to be preferred. Even if central fixation is not always maintained, it seems plausible to suggest that children will tend to make greater use of the clear central area for distance fixation and, at most, only use the lenslet‐covered areas in the lower part of the lens when performing near tasks, although this will be more common if the clear area is of smaller diameter.

As far as accommodation is concerned, informal reports suggest that, even when fixating through the lenslet‐covered areas of the lens, young wearers normally choose to bring the carrier image close to the retina, that is, they accommodate in the same way as they would with a single‐vision correction rather than making use of any add effect conferred by the lenslets. Measurements of near visual acuity through the lens‐covered areas of a MiyoSmart lens, a task demanding accurate accommodation, give results which are only marginally worse than those found with a matched single‐vision lens. This implies that accommodation is essentially the same under the two lens conditions.^18^, ^19^ This would be reasonable, since at the lenslet focus the lateral separation between the in‐focus images produced by different lenslets results in a confused image which is likely to be a poor accommodation stimulus. Thus, a child's foveal images during both distance and near observation might be expected to approximate to the similar ‘unaccommodated distance’ and ‘accommodated near’ images derived in the present paper, although there will be minor differences with object distance due to accommodation errors and small changes in ocular aberrations with accommodation.

On the peripheral retina, where the image is influenced generally by the effects of lenslets, the real‐life focus state is more ambiguous. It is very unusual for the dioptric accommodation stimulus provided by the visual environment to be constant across the visual field. Although such environments may be approximated under some outdoor situations, they are rare indoors, particularly when near tasks are being carried out.^20^, ^21^, ^22^ Accommodation is primarily optimised in terms of the foveal image so that an eye accommodated to foveal tasks is often over‐accommodated to peripheral parts of the field where objects are at greater distances from the eye.

Given these assumptions, two main hypotheses have been proposed to account for the apparent effectiveness of MS lenses in reducing any progressive increase in childhood myopia and axial length.^5^ The first hypothesis^2^ is that any observed reduction is a response to the creation in the more peripheral retina of two focal planes, one due to the basic carrier and the other, anterior to the retina, by the lenslets. The positions of the local image foci as calculated from our model when fixation of a distant object point is along the common lens/eye optical axis are shown in Figure 5. Note that this plot implies that fixation is axial, through the central clear area of the lens and it is assumed that accommodation is appropriate to distance vision. Effects vary with the field meridian considered.

As can be seen, in practice the image positions with respect to the retina are strongly affected by the astigmatism and other aberrations of the carrier and eye. There are not two unique ‘planes of focus’ but rather a ‘volume of focus’ in which some degree of focus occurs, which extends mostly, but not entirely, anterior to the retina. A similar effect is claimed for the Essilor Stellest MS lenses.^23^ The exact position of the images with respect to the retina may vary from that shown in Figure 6, depending upon the retinal shape of the individual. The important feature, however, is that the quality of any image of a distant object will vary asymmetrically about the carrier lens focus. The image formed by the carrier lens, with its marked central peak, gives a good image of any extended objects, although this is reduced in contrast compared with that formed by a single‐vision lens due to the out‐of‐focus light from the lenslets. Anterior to the retina, the in‐focus images formed by the lenslets will also provide higher spatial frequency information, but this anterior image is confused because of the lateral separation of the images formed by individual lenslets, making it of little value for general visual use. The asymmetry about the optimal (carrier) focus for the lenslet‐covered outer regions of the MS lens is well illustrated in the experimental model studies of Arias et al.^13^ However, when considering the relationship between image surfaces and the retina, it must be remembered that the present results are for a model with specific, ‘typical’ relative peripheral refractive errors. In practice, as noted earlier, children's relative peripheral refractive errors vary considerably, and there is no evidence that any specific form of these is linked to greater risk of myopia progression.^24^, ^25^ This, perhaps, weakens the argument that the effectiveness of MS lenses in slowing myopia development derives from the specific through‐focus changes in the point images in relation to the retinal surface that they produce.

The second hypothesis to account for the apparent effectiveness of MS lenses in controlling myopia progression, which receives great emphasis in the design of ‘diffusion‐optical technology’ (DOT) spectacle lenses,^26^ is the reduction in contrast that the lenses cause in the imagery of the peripheral field. As can be seen from the off‐axis plots like Figure 4C and the results of practical studies,^13^, ^14^ such contrast loss undoubtedly occurs with the MiyoSmart (DIMS) design. However, with the MiyoSmart over a range of focus, the minor peaks in the image structure associated with the light pencils passing through the small‐aperture (1 mm diameter) lenslets, with their large depth‐of‐focus, still ensure that some higher spatial frequency information is available to the retina. This structure is absent with DOT lenses,^26^ although they appear to reduce myopia progression to an extent similar to those found with the MiyoSmart design.^23^ It may be that it is image contrast loss in the peripheral retina that is the common factor of MS lenses that contributes most to the control of myopia progression, rather than the creation of an additional anterior plane of focus.^5^, ^27^ This concept is supported by the recent work of Su et al.,^28^ who found that the reduction in the rate of children's development of myopia and axial length was similar when lenslets of either negative or positive power were used. However, the hypothesis that reduced contrast in the peripheral field inhibits myopia progression appears to be in opposition to earlier views, based on animal studies, that any type of partial or total form deprivation in this area is a risk factor for such development.^21^, ^22^, ^29^ As yet, the mechanisms underlying any effectiveness of MS lenses are imperfectly understood.

It is important to recognise that a limitation to our modelling is that the retinal images for lens–eye combinations (Figures 4 and 6) were calculated using an adult model eye, whereas the MS spectacle lenses would normally be worn by children. An adult, rather than a child, model was chosen because the basic data underlying this model are much more reliable and complete than the corresponding data for children. For example, little is known about such factors as the asphericity of surfaces and retinal shape in the young eye. Moreover, ocular growth throughout childhood means that any full study of imagery with young lens wearers demands the use of multiple eye models. Overall, it appears unlikely that the general optical outcomes of the obliquity of ray pencils change markedly with age.

Part 2 of this paper will investigate the power corrections of the Hoya MiyoSmart lens in the peripheral (static) vision and foveal (rotating eye) situations.

## CONCLUSIONS

Multisegment spectacle lenses are thought to slow the progress of myopia in children by modifying the through‐focus image quality in the peripheral retina. Attempts to understand this process need to consider the way in which the through‐focus quality changes with the obliquity of the ray pencils across the visual field, since such pencils dominate imaging in the periphery. The present results show that the off‐axis aberrations of both the lens and eye, particularly peripheral astigmatism, reduce the quality and contrast of images formed on the peripheral retina. This degradation increases generally with field angle, although exact effects also depend upon the eye's fixation direction with respect to the optical axis of the MS lens. Rather than the carrier lens and lenslets giving two distinct planes of focus in the periphery, there is an extended region of approximate focus limited primarily by increasing astigmatism, and it seems probable that MS lens wearers usually accommodate to bring the carrier focus onto the retina. Under these circumstances, any myopia progression control effect seems more likely to be due to a lenslet‐induced loss of contrast in the peripheral retinal image rather than to the existence of two planes of focus.

## AUTHOR CONTRIBUTIONS

**W. Neil Charman:** Conceptualization (equal); investigation (equal); writing – original draft (equal). **David A. Atchison:** Investigation (equal); methodology (equal); resources (equal); writing – original draft (equal). **Matt Jaskulski:** Methodology (equal); writing – review and editing (equal).

## FUNDING INFORMATION

None.

## CONFLICT OF INTEREST STATEMENT

Neil Charman: None. David Atchison: None. Matt Jaskulski: Co‐founder and CEO of VisionApp Solutions S.L. which is a start‐up company developing mobile software for vision.
